# The Emerging Role of Long Non-Coding RNAs in Plant Defense Against Fungal Stress

**DOI:** 10.3390/ijms21082659

**Published:** 2020-04-11

**Authors:** Hong Zhang, Huan Guo, Weiguo Hu, Wanquan Ji

**Affiliations:** 1 State Key Laboratory of Crop Stress Biology for Arid Areas, College of Agronomy, Northwest A&F University, Yangling 712100, China; guohuan2018@163.com (H.G.); hnhuweiguo@163.com (W.H.); 2 Shaanxi Research Station of Crop Gene Resource & Germplasm Innovation, Ministry of Agriculture, Yangling 712100, China

**Keywords:** lncRNAs, plant immunity, transcriptional regulation

## Abstract

Growing interest and recent evidence have identified long non-coding RNA (lncRNA) as the potential regulatory elements for eukaryotes. LncRNAs can activate various transcriptional and post-transcriptional events that impact cellular functions though multiple regulatory functions. Recently, a large number of lncRNAs have also been identified in higher plants, and an understanding of their functional role in plant resistance to infection is just emerging. Here, we focus on their identification in crop plant, and discuss their potential regulatory functions and lncRNA-miRNA-mRNA network in plant pathogen stress responses, referring to possible examples in a model plant. The knowledge gained from a deeper understanding of this colossal special group of plant lncRNAs will help in the biotechnological improvement of crops.

## 1. Introduction

Long non-coding RNAs (LncRNA), defined as a group of RNA transcripts that exceed 200 nt in length with no apparent discernable coding potential, were transcribed from the non-functional gene regions in eukaryotes. The recent explosion in knowledge has demonstrated that lncRNAs should be key regulators of the protein-coding gene expression levels, directly or indirectly [1,2]. Compared with mammal research, the functional dissection of plant lncRNAs is far behind. Luckily, the identification of plant lncRNAs has largely caught up with the mammalian field over the last few years, especially using high-resolution analyses of plant transcriptomes, which also allowed a more comprehensive view of lncRNAs in plants. Emerging evidence indicates that lncRNAs play key roles in diverse biological processes in plant development, including flowering [3], root organogenesis, seedling photomorphogenesis [4], reproduction, and defense against fungal infections [5]. According to the general location, long ncRNAs were classified into long intron ncRNAs, promoter lncRNAs, long intergenic ncRNAs (lincRNAs), and natural antisense transcripts (lncNATs) [6]. The natural antisense transcript could target mRNA and form an RNA dimer via complementary base pairing, and finally block the binding sites of transcription factors in humans [7,8,9]. Some lncRNAs bind miRNAs and competitively inhibit the interaction between miRNAs and target mRNAs to modulate gene expression [10,11,12]. LincRNA transcription appears to positively or negatively affect the expression of nearby genes, although not all are active [13,14]. Thus, lncRNAs in plants can be considered as essential elements of gene regulation and as a potential resistant gene resource. Plants are sessile and must continuously adapt to occasional and inevitable environmental coercion, not only in abiotic but also biotic signals during their life cycle. For example, stripe rust (*Puccinia striiformis* f. sp. *tritici*; Pst) and powdery mildew (*Blumeria graminis* f. sp. *tritici*; Bgt) are two important fungal diseases of wheat (*Triticum aestivum*) in the world, and both result in significant crop damage in epidemic years [15], threatening the safety of our food supply. So, understanding the resistance mechanism can undoubtedly be of benefit in controlling disease and minimizing crop losses. Because plants lack circulating cells, they rely on systemic signals emanating from infection sites to trigger innate immunity, especially on phytohormones. The phytohormone-treated wheat transcriptome revealed that about 92% of abscisic acid (ABA)-responsive genes were similarly expressed after *Fusarium graminearum* pathogen infection [16]. In this process, thousands of genes are implicated, including functional genes and also noncoding RNAs (ncRNAs) encoded by a specific genome region [17], which account for 90% of the genome [18]. A large-scale sequencing analysis revealed that most of the eukaryotic genome is transcribed to RNAs, including short and long ncRNAs [5,19,20]. However, detailed information of the lncRNAs functioning in plant defense has not been very well summarized. Therefore, a large number of data on the direct and/or indirect interaction between mRNA and lncRNA related to plant immunity are of vital significance for an in-depth study of the functional mechanism of lncRNAs in plant. Here, we describe the major identification of plant lncRNAs, and how they act, with a focus on research in crops and our emerging understanding of lncRNA functions in serving as microRNA precursors, molecular sponges, and decoys, functioning in the regulation of transcription and silencing, particularly in alternative splicing, and epigenetic regulation of the defense against fungal disease. It is hoped that lncRNAs will be exploited as a mainstream player to achieve food security by tackling different plant diseases.

## 2. Capturing LncRNAs in Plant

The literature investigating the role of lncRNAs in various biological processes of plants has increased over the last 10 years [19,21,22]. These efforts have identified a myriad of molecular functions for lncRNAs (Table 1). From >200 transcriptome data sets in *Arabidopsis*, ~40,000 candidate lncRNAs were identified [23], including NATs (>30,000) [24] and lincRNAs (>6000) [25]. The statistics showed that ~70% of the protein-coding loci in *Arabidopsis* transcribed candidate NAT pairs from an opposite strand. Some NAT pairs show complete overlap (~60%), but others have complementary segments at their 5′ or the 3′ ends. LncRNAs were enriched and diversified in crop plants because of the effect demonstrated in *Arabidopsis*. A total of 2542 lincRNA candidates in *Populus trichocarpa* (*P. trichocarpa*) [26], nearly 10,000 lncRNAs in maize (*Zea mays* L.) [27,28,29,30], as well as 682 high-confidence lncRNAs in cassava [31] were identified. In rice, by performing whole-transcriptome strand-specific RNA sequencing (ssRNA-seq), 2224 lncRNAs involved in the reproductive process [32] and 2588 novel putative lncRNA encoding loci under nitrogen starvation were verified [33]. In *Brassica*, 1885, 1910, and 1299 lncRNAs at the whole genome level were identified for *B. napus*, *B. oleracea*, and *B. rapa*, respectively [34]. Mining the Camelina (*Camelina sativa* L.) drought-stressed cDNA library, 5390 candidate CsalncRNAs were identified, including 670 sense, 692 antisense, 1347 intergenic, and 2681 intronic lncRNAs [35]. During tomato resistance to *Phytophthora infestans* infection, 9011 lncRNAs were identified from tomato plants [36]. In hexaploid wheat, polyadenylated lncRNA and lncNAT were captured using a low-efficiency Race experiment [37] or high-though sequencing technology [5,38], while they were also reported to play a role in wheat responding to fungal infection. Notably, fungi-responsive lncRNAs in wheat were identified and their function predicted using genome-wide microarray analysis, SBS sequencing, and RNA-Seq data [5,39]. Using the RNA sequencing (RNA-Seq), Zhang et al. identified 58,218 lincRNAs from wheat seedlings and predicted the function of 283 DE-lincRNA implicated in the interaction of wheat with fungi [5]. These studies of lncRNA lay the foundation for an investigation of the functions of lncRNAs and the defense mechanism of plants against fungi.

## 3. LncRNAs in Plant Defense against Fungal Stress

Plants resist diseases using two major types of immune receptors, receptor-like kinases or receptor-like proteins (RLKs/RLPs) and disease resistance (R) functional proteins, which are responsible for pathogen recognition and subsequent defense induction. The mechanism of plants’ defense against pathogens has been very well reviewed and visualized using models [51,52,53]. In this feedback control loop, all of the implicated functional genes and their regulators’ expression suffer from transcriptional and post-transcriptional regulation [54]. LncRNAs are present at low levels and show little sequence conservation compared with mRNAs; therefore, early studies suggested that lncRNAs might result from transcriptional noise. However, evidence has emerged to indicate that many lncRNAs function in a large number of diverse molecular processes in eukaryotic cells. In plants, lncRNA functions were mainly reported in photomorphogenesis in seedlings, organogenesis in roots, flowering time control, abiotic stress responses, and reproduction, as aforementioned. Recently, because of the importance of defending regulation for plant adaptation to different pathogens, lncRNAs that regulate resistance are also constantly being discovered in plants, although the number of related reports is less than other aspects. This characterization could be summarized into three general expression and construct properties. The first is that many lncRNAs are not only polyadenylated and capped but also non-polyadenylated. The second is that lncRNAs tend to be expressed at lower levels than protein-coding genes, but with precise spatio-temporal patterns [55,56]. Thirdly, some lncRNAs contain parts of an exon, while a proportion of lncRNAs are derived from transposable elements (TEs) or contain remnants of TEs [57,58]. Here, we focus on the advance and regulatory function in plants responding to fungi underlying these properties and recent research on lncRNAs.

### 3.1. Responding to Biotic Stress and Co-Expression with Functional Genes

Since some lncRNAs of the model plant, *Arabidopsis thaliana*, have been proven to be involved in the response to *Fusarium oxysporum* infection [40], a large number of lncRNA functions in plant–fungi interactions have been reported in succession (Table 1). After early infection by *Melampsora larici-populina*, a total of 3994 lncRNAs were identified by mining the RNA-Seq data, and then 53 differentially expressed lncRNAs (DE-lncRNAs) were detected in poplar between treatments and controls [47]. About 63 DE-lncRNAs in maize roots responding to *Arbuscular mycorrhizal* fungi were predicted to regulate the allelic-specific protein-encoding genes as a cis-regulator or miRNA mimic [45]. It is noted that the putative target genes of differentially expressed lncRNAs (DELs) in maize were mainly related to phosphate ion transmembrane transport, cellular response to potassium ion starvation, and lipid catabolic processes. In melon, 611 lncRNAs were found to be differentially expressed after powdery mildew infection in a PM-resistant melon line [46]. Additionally, by comparing the transcriptome, tomato lncRNA16397 was demonstrated by inducing *SlGRX* expression to reduce ROS accumulation, thereby enhancing resistance to *Phytophthora infestans* [49]. From 931 DE-lncRNAs detected in rape infected with *Sclerotinia*, 9 lncRNAs showed overlap with cis-regulatory regions of DEGs of *B. napus*, while an lncRNA, numbered TCONS-00000966, exhibited 90% overlap with plant defense genes [43]. Meanwhile, the current transcriptomic analysis identified lncRNAs associated with *Fusarium* head blight (FHB) resistance [42]. The time-course RNA-seq analysis discovered 559 lincRNAs in response to *Pectobacterium carotovorum* subsp., and 17 of these lincRNAs were found to be highly co-expressive associated with 12 potato defense-related genes [48]. Similarly, 514 lncRNAs in resistant *Gossypium barbadense* were identified as species/lineage-specific (LS) lncRNAs involved in the resistance to *Verticillium dahliae*, a fungal disease in cotton [44]. Further functional analysis showed that GhlncNAT-ANX2- and GhlncNAT-RLP7-silenced seedlings displayed an enhanced resistance towards *V. dahliae* and *Botrytis cinerea*, possibly associated with the increased expression of lipoxygenase 1 (*LOX1*) and lipoxygenase 2 (*LOX2*) [44]. A long intergenic noncoding RNA, *LINC-AP2*, is upregulated and negatively correlated with *AP2* gene expression with *Turnip crinkle* virus infection in *Arabidopsis* [59]. Work in *Arabidopsis* has also shown that the intron lncRNA, COLDAIR, regulates the initiation of flowering by modulating the expression of *FLOWERING LOCUS C* (*FLC*), which encodes a MADS-box transcription factor [3]. In hexaploid species, although the fungi-responsive lncRNAs and LncNAT were reported as early as 2011 [37,39], the research is far slower than in diploid species due to a delay of the reference genome’s publication. So far, about 400 DE-lncRNAs have been predicted in wheat infected by *Pst* and *Bgt*. Transcription factors (TFs) are important regulators of gene expression in plants responding to abiotic and biotic stress. The NAC, WRKY, AP2/ERF, and C2H2 TFs have been reported to be involved in plants’ response to pathogens [60]. Analyzing the co-expression of lncRNAs with NAC17L, NAC68L, WRKY55L, C2H2, and WRKY64/70, the results showed that lncRNAs have a co-regulation relationship with neighboring TF genes, although it exhibited an opposite and/or positive expression pattern in a differential genetic background [61]. These results hinted that lncRNAs play regulatory roles, resulting in co-expression with adjacent protein-coding genes, in both positive and negative ways.

### 3.2. Regulation of LncRNAs’ Defense Against Fungal Stress though miRNA and siRNA Precursor

Although plant long noncoding RNAs (lncRNAs) have now increasingly been shown to be involved in biological processes as regulatory molecules, the functional role of many of the members has still been an enigma in plants. Recent reports have suggested that lncRNAs could potentially interact with other classes of non-coding RNAs, including microRNAs (miRNAs) [62,63,64,65,66,67]. MicroRNA are endogenous short ncRNAs (21–24 nucleotides) that play important regulatory roles by repressing gene translation or degrading target mRNAs at the post-transcriptional levels [19,68]. By mapping miRNAs to 125 lncRNAs, Xin et al. [39] identified four transcripts and characterized microRNA (miRNA) precursors (TalnRNA5, TapmlnRNA8, TapmlnRNA19, TahlnRNA27), which showed stable hairpin structures. Among the four long npcRNAs, TalnRNA5, TapmlnRNA19, and TapmlnRNA8 were responsive to powdery mildew infection [39]. By comparing with small RNA-seq data, Li et al. [31] found 42 lncNATs and sense gene pairs can generate nat-siRNAs in cassava. Similarly, Gao et al. [46] also identified 24 lncRNAs that act as microRNA (miRNA) precursors in melon responding to powdery mildew. As for hostility, tomato yellow leaf curl virus (TYLCV) also employs an intergenic ncRNA (siRNA)-targeting host lncRNA *SlLNR1* in TYLCV-susceptible tomato to modulate disease symptoms [69]. These results give cues that lncRNAs play a role in regulating functional genes as the precursors of miRNA or siRNA.

### 3.3. Regulation of Defense against Fungal Stress as Molecular Sponges and/or Decoy

Apart from the role of a regulator as miRNA precursors, lncRNAs are also potential targets and endogenous target mimics (eTMs) of miRNAs in plants. For example, out of 466 lincRNAs that were considered as 165 miRNA targets, 86 lincRNAs were thought to be 58 miRNA decoys in maize [70]. In total, 16 lncRNAs were identified as putative target mimics of cassava’s known miRNAs [31]. Gao et al. [46] identified 43 lncRNAs that act as potential targets and 13 lncRNAs as endogenous target mimics (eTMs) in melon. Similarly, slylnc0195, slylnc1077, and 14-nt-deleted *SlLNR1* are found to act as decoys for miRNAs in tomato against TYLCV [50,69], while several lncRNA were found to modulate MYB, HD-Zip, and NAC transcription factors in response to *Phytophthora infestans* infection by decoying miR159, miR166b, and miR164a-5p, respectively [36]. Moreover, LncRNA39026 enhances tomato resistance to *P. infestans* by decoying miR168a and inducing PR genes’ expression [71]. lncRNA directly binds to protein mediator as a molecular decoy of FIBRILLARIN 2 (FIB2) to regulate gene transcription of ELF18-induced long noncoding RNA1 (*ELENA1*) regulating PR1 in *Arabidopsis* [72]. After *Bgt* and *Pst* infection in wheat, 101 lncRNAs were predicted to be the target of miRNA using psRNATarget, including miR156, miR160, miR164, miR167, miR393, miR398, miR829, and so on. Moreover, five target mimics were identified form DE-lncRNAs responding to fungi, which target tae-miR167a, ath-miR390a, ata-miR156d-3p, ata-miR160a-3p, ath-miR394a, ata-miR395c-5p, and ath-miR399b [5]. Subsequently, the Q-PCR results showed that the gene expression level of lncRNA T13.17661 and TraesCS1B02G415800.1 (both targeted by miR399b) were low and stable after *Bgt* inoculation, but the expression of miRNA399b was upregulated 4- to 7-fold compared to 0 hpi in a resistant background [61]. Regardless of whether miRNA regulatory elements cluster towards the mid regions and 3′ ends of long noncoding transcripts like in Zebrafish [62], this provides a cue that lincRNA could competitively interplay with functional genes via miRNA regulation. In a word, although the experimental data on miRNA and lncRNA in the resistant field of plants is still insufficient, these data substantiated that lncRNAs could participate as a layer of regulatory interactions with miRNAs, and are thus implicated in plant immunity to fungi. 

### 3.4. Regulation of LncRNAs as ceRNA though Pseudogene Transcripts

Pseudogenes are degenerate copies of genes that are synthesized mostly through DNA duplication (duplicated pseudogenes) and retrotransposition of cellular RNAs (processed pseudogenes) [73]. Although the potential for pseudogene transcripts to encode proteins is acknowledged [74], the majority of pseudogenes should be considered as lncRNAs due to accumulated mutations causing frame shift or premature stop codons mutations. As transcribed pseudogenes commonly share miRNA-binding sites with their parent genes, they are considered attractive candidates as competitive endogenous RNA (ceRNAs). We expect such examples to emerge for plant lncRNAs in the future.

### 3.5. The Potential for LncRNAs to Influence Functional Genes via Alternative Splicing

LncRNAs in mammalian cells have a role of binding and sequestering serine/arginine (SR) splicing factors, leading to an altered pattern of alternative splicing (AS) transcripts for pre-mRNAs. LncRNAs can also regulate intron splicing of the sense transcripts by masking splicing sites through its complementary sequences [75,76]. AS has been very well substantiated to alter the transcripts of related genes under biotic stresses [54,77]. In plants, alternative splicing competitor lncRNA (ASCO-lncRNA) can hijack nuclear speckle RNA-binding protein (NSR) to alter the splicing patterns of transcripts in response to auxin [76]. Transcriptional analysis of hormone responses in wheat showed that 5 SA-, 85 MeJA-, 718 ABA-, and 23 ET responsively specific DEGs were similarly expressed with defense-related genes responding to *F. graminearum* infection, and the expression of some DEGs were also similar following fungal stress with IAA treatments [16]. Several lncRNA target genes were identified and annotated in plant hormones, which suggests lncRNAs can regulate the metabolism and signal transduction of plant hormones [78]. Zhang et al. [5] analyzed the SnRNP motifs of DE-lncRNAs in wheat after *Pst* and *Bgt* infection. A total of 1328 SnRNP motifs were detected from 246 differential expressed lncRNAs. These motifs were further classed into 47 putative Sm-sites and 407 SnRNA oligonucleotides. The Sm-site element of spliceosomal snRNA is characterized by a consensus RRU_4–11_RR construct. The Sm-site element of U1, U2, U4/6, and U5 spliceosomal snRNA is characterized with consensus PuAU_3–6_GPu in mammalian genomes. Recently, U1 snRNP was thought to regulate chromatin retention of noncoding RNAs due to the asymmetric distribution of splicing sites at the 5′ and 3′end of lncRNA [79]. This hinted that lncRNA play a critical role in AS of plant functional genes related to R and/or phytohormones signals responding to fungal infection.

## 4. Regulation Mechanism of LncRNAs in Gene Transcription

**Figure 1:**
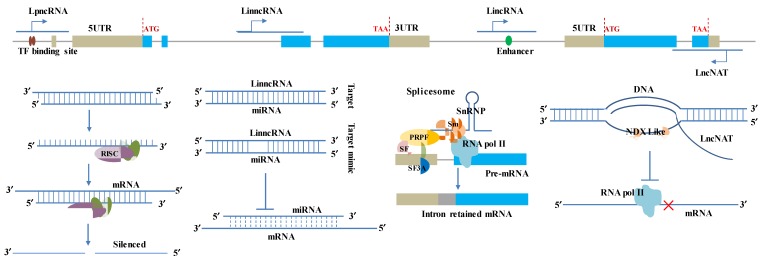
LncRNA Schematic illustrations for classification and regulation mechanism in functional genes. The lncRNA classes were given on the top based on the DNA sequences and cDNA sequences of two simulated tandem genes. Open reading frames (ORFs) and exons are shown as blue boxes. The introns of the genome sequences are indicated by grey lines. Untranslated regions (UTRs) are shown as beige boxes. The regulation mechanism of lncRNA in functional genes was simulated in the bottom panel, including miRNA precursors, competitor/decoy (miRNA target/target mimic), alternative splicing, and R-loop. The dash lines represent the alignment probability between miRNA and mRNA was reduced due to competition by lncRNAs. The outline and shapes of spliceosome complexes tethering lncRNA are purely schematic; their size and number have no meaning. ‘Sm’ indicates the seven Sm proteins, i.e., B, D1, D2, D3, E, F, and G; ‘PRPF’ and ‘SF’ mean pre-mRNA processing factor and splicing factor; snRNPs contain the indicated lncRNA (containing an Sm site bound to RNA-binding protein) plus stably bound proteins, many of which are shared with the major spliceosome. SnRNP association with RNA polymerase II in the spliceosome has only been inferred to date. The regulated genes could be any potentially functional gene, including pathogen perception and signal transduction-related genes, R genes, their regulator (transcription and splicing factors), and so on.

In the nucleus, lncRNA may execute its function either in cis or in trans. It has been proposed that lncRNAs transcribed at a low level are likely to work in cis, whereas those that accumulate at a higher level are able to act in trans. There are many possible ways for lncRNAs to regulate the functional gene’s transcription machinery (Figure 1). For example, animal lncRNAs can regulate the DNA-binding activity of TF by modulating TF dimerization or trimerization, promoting TF phosphorylation or controlling TF nuclear localization [80,81,82]. LncRNAs that are transcribed from the enhancer domains and/or transcription factor binding sites of genes can act as transcriptional coactivator/repressors and/or to control chromatin topology [83,84,85] to regulate the transcription activities of their flanking genes [75]. Moreover, lncRNAs can also interact with mediator subunits and regulate mediator complex formation [86,87]. One mode of lncRNA action is to trigger the formation of a stable RNA–DNA triplex to control TF binding specificity on promoter regions [88,89,90,91]. In *Arabidopsis*, *COOLAIR*, an antisense transcript originating from the 3′ end of the floral repressor gene *FLC*, can reconfigure double-stranded DNA to a RNA–DNA hybrid and a single-stranded DNA (ssDNA) called ‘R-loop’ [92]. The released ssDNA is bound by the homeodomain transcription factor, AtNDX, which stabilizes the R-loop and inhibits *COOLAIR* transcription. Differential stabilization of lncRNA and R-loops is likely to be a common mechanism controlling the transcriptional activities on many genes [93,94]. Recently, AtNDX was considered to interact with the polycomb repressive complex1 (PRC1) core components AtRING1A and AtRING1B, negatively regulating the expression levels of ABA-insensitive *ABI4* by targeting the end of *ABI4* [95]. Indeed, the B3 domain transcription factor ABI3, APETALA2-type transcription factor ABI4, and bZIP transcription factor ABI5 functioned genetically in the downstream of AtNDX. ABA-mediated growth arrest in *A. thaliana* was controlled by histone demethylases [96]. The expression of *ABI3* increased in ndx, but AtNDX could not directly bind to *ABI3* [95]. Considering all the aforementioned information together, we can infer that AtNDX may regulate the expression of *ABI3* though the lncRNA regulator *COOLAIR* by acting in *FLC*.

## 5. Molecular Network of Plant LncRNAs Related to Resistance

So far, no detailed functional network studies of plant lncRNA have been reported, especially in plants responding to pathogens. However, 1077 DE-lncRNA were predicted in maize during abiotic stress, like heat, cold, salt, and drought. Studies inferring co-expression networks revealed that 39 lncRNAs are major hubs in the co-expression networks, and 18 lncRNAs were considered to be derived from long-terminal-repeat transposable elements (TEs) [57]. A ceRNA network was constructed with the identified DElncRNAs, DEmiRNAs, and DEmRNAs in the roots of Ziyang Xiangcheng (*Citrus junos*) under Cu toxicity [97]. These results suggest that lncRNAs, including TE-lncRNAs, may play key regulatory roles in moderating plant abiotic responses via the systemic molecular network. Recently, the gene co-expression level of lncRNA T13.17661, an Ub-enzyme E2 gene, and their target miRNA399b were analyzed in *Bgt*-infected wheat leaves using Q-PCR [61], which substantiated the view that lincRNA could competitively interact with mRNA via miRNA regulation. Additionally, the availability of functional cues for plant lncRNAs across the transcriptome has prompted us to reconstruct a genome-scale network of interactions between mRNA, TFs, miRNAs, and lncRNAs though computational science. For example, CIRNN, the ensemble deep-learning model based on convolutional neural network (CNN) and independently recurrent neural network (IndRNN) [66], plus SIMCLDA [98] will help us to accelerate the dissection of the interaction between the miRNA and lncRNA of plants. In wet experiments, CLIP-Seq methods have reliably identified argonaute (Ago) and other RNA-binding protein (RBP) binding sites to characterize miRNA–mRNA and lncRNA–miRNA interactions [99]. Taken together, we will understand more interactions between lncRNA, miRNA, TF, and mRNA, and then construct the network of lncRNA-TF–mRNA and lncRNA–miRNA–mRNA.

## 6. Prospect of Plant LncRNAs Related to Resistance

Unfortunately, most of the aforementioned molecular sponges and/or decoys were based on bioinformatics analysis. By contrast, only a small number of lncRNAs related to plant immunity have been functionally characterized so far. However, the report that non-coding RNA *IPS1* (*INDUCED BY PHOSPHATE STARVATION1*) from *A. thaliana* alters the stability of *PHO2* mRNA by sequestering the phosphate starvation-induced miRNA miR-399 [10] gives the first successful example of functional lncRNA in plants. Another example is that the long noncoding miRNA gene *Iw1* (*INHIBITOR of WAX1*) targets *W1-COE*, repressing wheat β-diketone waxes’ synthesis [100]. Similarly, the linncRNA *COLDAIR* (cold-assisted intronic noncoding RNA) and lncNAT *COOLAIR* are related to the vernalization-mediated epigenetic repression of *FLC* [3]. The *ELF18-INDUCED LONG-NONCODING RNA1* (*ELENA1*), as a factor enhancing resistance against *Pseudomonas syringe* pv. *tomato* DC3000, directly interacts with FIB2 and mediator subunit 19a (FIB2/MED19a) and affects the enrichment of MED19a on the *PR1* promoter [41,72]. Overexpressing lncRNA *LAIR* was proven to increase grain yield and regulate the neighboring gene cluster’s expression in rice [14]. Although most of the experiments focused on plant development, it is no doubt that these works give confidence to the researcher in finding functional lncRNAs in plants responding to pathogen stress. 

## 7. Concluding Remarks

Plants, unlike animals, rely on systemic signals emanating from infection sites to trigger the innate immunity through resistance (R) genes. The R genes of plants are frequently overcome by epidemic pathogens. Understanding the resistance mechanism could undoubtedly be of benefit in controlling disease and minimizing crop losses. In this process, thousands of genes are implicated, including lncRNAs. The association of long non-coding RNAs with plant immunity stimulated researchers to exploit lncRNA regulator machinery in terms of augmented plant immunity against diseases. However, the characterization of lncRNAs’ function in plants remains limited. Although considerable efforts are being performed globally to recognize the protection purpose of plant lncRNAs, the precise anti-pathogenic defense part of their function is lacking. In fact, the lack of lncRNA sequence similarity will certainly challenge the identification of homologous lncRNAs in crops. Hopefully, the pace of identifying ‘functional’ lncRNAs will be accelerated using synteny regions between a crop plant and its corresponding model plant. Here, we have summarized long noncoding RNAs in plants infected with fungi, and then evaluated the possible regulating role of lncRNAs on functional genes. The results hinted that lncRNAs play the potential role of regulating the allele-specific gene, including transcription factors. These results will be beneficial for further dissecting the molecular mechanisms of lncRNAs’ functions at the transcriptional and post-transcriptional levels in plants, especially the role in plant–fungi interaction, because pathogens may also have enhanced virulence by the development of lncRNAs. Lastly, we provided an overview of the emerging techniques and databases that are employed for the identification and characterization of plant lncRNAs, which will facilitate further investigations into different types of resistance-related genes of crops in the future. We believe lncRNAs will enrich resistance gene resources, like TF genes, for the biotechnological improvement of crops, because the evidence of lncRNAs’ multiple regulatory functions has been given in model plants. In conclusion, we expect that further research on the lncRNA-mediated plant defense against pathogens, by achieving disease resistance, will help scientists enhance global food security.

## Figures and Tables

**Table 1:** List of long non-coding RNAs (lncRNAs) identified in plants under fungal pathogen stress.

Plant Species	Stress/Response	Approaches	Type(s) of LncRNAs	LncRNAs Number	DE-LncRNAs Number	Ref.
***Arabidopsis***	light	tiling array	LncNATs	37,238	1392	[24]
	drought, cold, high-salt, ABA	tiling array&RNA-seq	LincRNAs	6480	1832	[25]
	*Fusarium oxysporum*	ssRNA-seq	LncNAT, TAR	2346/770	15/25	[40]
	*Pseudomonas syringae*	In silico-EST	LincRNAs	1(*ELENA1*)	1	[41]
**Barly**	*Fusarium graminearum*	RNA-seq	LncRNAs	12,366	604	[42]
***Brassica napus***	*Sclerotinia sclerotiorum*	RNA-seq	lincRNAs	3181	931	[43]
	-	RNA seq	LncRNAs	1885	-	[34]
***Camelina sativa***	drought	FlcDNA seq	LncRNAs	5390	7	[35]
**Cassava**	cold, drought	ssRNA-seq	LncRNAs, lncNATs	682/42	318	[31]
**Cotton**	*Verticillium dahliae*	RNA-seq	lincRNAs, LncNATs	13,452/1297	1236/63	[44]
**Maize**	*Rhizophagus irregularis*	ssRNA-seq	LncRNAs	9541	63	[45]
	-	EST, Gen-seq&RNA-seq	LncRNAs	1704	tissue-specific	[27]
	nitrogen	RNA-seq	lincRNAs, linncRNAs	7245	637	[28]
	drought	RNA-seq	LncRNAs	1724	664	[29]
	-	FlcDNA seq	LncRNAs	2492	-	[30]
**Melon**	*Podosphaera xanthii*	RNA-Seq	LncRNAs	11,612	611	[46]
**Poplar**	*Melampsora larici-populina*	RNA-seq	LncRNAs	3994	53	[47]
***Populus trichocarpa***	drought	RNA-Seq	LincRNAs	2542	504	[26]
**Potato**	*Pectobacterium carotovorum*	ssRNA-seq	LincRNAs	1113	559	[48]
**Rice**	sexual reproduction	ssRNA-seq	LincRNAs, lncNATs	1624/600	tissue-specific	[32]
	Nitrogen, phosphate starvation	ssRNA-seq	LncRNAs	2588	776	[33]
**Tomato**	*Phytophthora infestans*	RNA-Seq	LncRNAs	28,256	688	[49]
	*Tomato yellow leaf curl virus*	ssRNA-seq	LincRNAs, lncNATs	1289/276	529	[50]
**Wheat**	*Blumeria graminis* (*Bgt*)	Microarray&RNA-Seq	LncRNAs	125	52	[39]
	*Puccinia striiformis* (*Pst*)	In silico-EST	LncRNAs, NAT	3/1	3/1	[37]
	*Bgt*, *Pst*	RNA-Seq	LncRNAs	58,218	254/52	[5]
	heat, drought and salt	RNA-Seq	LncRNAs	44,698	413/14,162	[38]

Note: TAR, transcriptionally active regions; lincRNA, long intergenic ncRNAs; linncRNA, long intron ncRNAs; lncNAT, natural antisense transcription.
